# *Notes from the Field:* Measles Outbreak — Cook County, Illinois, October–November 2023

**DOI:** 10.15585/mmwr.mm7310a3

**Published:** 2024-03-14

**Authors:** Kelley Bemis, Mabel Frias, Sheila Giovanni, Tarek Shackour, Heather D. Reid, Jodi Morgan, Michael TeKippe, Demian Christiansen

**Affiliations:** ^1^Cook County Department of Public Health, Forest Park, Illinois; ^2^Illinois Department of Public Health; ^3^Advocate Children’s Hospital, Oak Lawn, Illinois.

**Summary**

**What is already known about this topic?**

Measles is a highly contagious vaccine-preventable disease. In the United States, 2 doses of measles, mumps, and rubella (MMR) vaccine are recommended for all children aged 12–15 months and 4–6 years.

**What is added by this report?**

During October 5–November 1, 2023, five measles cases occurred in unvaccinated, vaccine-eligible children aged 1–9 years who lived in the same apartment building but did not socialize with one another. During the outbreak, approximately 400 persons were exposed to measles, including 13 children aged <1 year.

**What are the implications for public health practice?**

Two doses of appropriately spaced MMR vaccine are recommended for all children and other susceptible persons to prevent measles cases and outbreaks.

On October 10, 2023, the Cook County Department of Public Health (CCDPH) in Illinois was notified by hospital A, a large pediatric facility, of a suspected measles case in a child aged 2 years (patient A) who had immigrated from Yemen on September 29 and who had no history of receipt of measles, mumps, and rubella (MMR) vaccine. The child visited hospital A’s emergency department (ED) on October 5 with fever, cough, and coryza and, after receipt of negative COVID-19, influenza, and respiratory syncytial virus test results, received a diagnosis of an unspecified viral illness. On October 8, the child visited hospital B’s ED with worsening respiratory symptoms and received a positive rhinovirus/enterovirus test result on a respiratory pathogen panel, after which the child was transferred to hospital A and admitted for respiratory distress related to bronchiolitis and underlying reactive airway disease. The next day, while hospitalized, the child developed a maculopapular rash. On October 10, the child’s family reported contact with a person with clinically diagnosed measles before U.S. arrival.* Measles was confirmed by real-time reverse transcription–polymerase chain reaction (RT-PCR) testing on October 11; the child was discharged the same day.

## Investigation and Outcomes

During the child’s October 5–11 health care encounters, 247 health care workers^†^ and 177 patients and patient companions^§^ were considered to have been exposed, including 13 children aged <1 year, five immunosuppressed children, and one child aged >1 year with no history of MMR vaccination. Among these 19 children, two received a dose of MMR vaccine within 72 hours of the exposure, and 13 received immune globulin.

The index patient’s household contacts included two siblings with no history of MMR vaccination and with serologic testing indicating measles susceptibility. One sibling, aged 4 years, (patient B) arrived in the United States at the same time as the index patient (September 29). The second sibling, aged 9 years, (patient C) had arrived in the United States in January 2023. Both siblings developed measles while in quarantine with rash onsets on October 22 (patient B) and November 1 (patient C). Patient B also reported fever, cough, coryza, and conjunctivitis; patient C also reported fever. Neither child was hospitalized, although patient B required an ED visit at hospital A for supportive care. On October 17, exposure notification letters were delivered to all residents in the apartment building where the index patient lived.

On October 30, hospital A notified CCDPH of another child, aged 2 years, (patient D) who had been evaluated in an ED early that morning with fever, cough, and coryza, then discharged. The family of that child lived in the same 2-story apartment building as the index patient, but on a different floor. Patient D had no history of MMR vaccine; the child’s parents reported objections to MMR vaccine based on personal beliefs and perceptions about vaccine side effects. Measles was confirmed in this child by RT-PCR testing on October 30; rash onset occurred on November 1. The families of patients A–C and patient D had different cultural backgrounds from one another and spoke different primary languages; both families independently reported no contact with the other family. Their apartment units did not have shared ventilation; however, laundry facilities and building entrances were shared.

On October 31, testing was also performed for a sibling of patient D, a child aged 1 year (patient E), also with no history of MMR vaccine, who had isolated coryza and who attended a child care facility^¶^ on October 30 while symptomatic; a nasopharyngeal swab collected in the home confirmed measles by RT-PCR testing. Attendees and staff members of the child care facility were notified the same day. One child aged 2 months received immune globulin, one child aged 11 months received 1 dose of MMR vaccine, and 11 children who had received their first MMR vaccine dose received an early second dose as post-exposure prophylaxis.** Fever in patient E did not occur until November 6, and rash did not appear until November 9, which was 9 days after the positive RT-PCR test result and child care facility notification.^††^ Measles testing is indicated for susceptible contacts of measles cases when the contact has prodromal symptoms (i.e., fever, cough, coryza, or conjunctivitis); however, isolated coryza experienced by this patient at the time of specimen collection might not have been related to measles. Because testing for measles before fever onset is not typically performed, an accurate infectious period for this patient was difficult to ascertain. Patient E’s symptoms resolved without requiring emergency care or hospitalization. This activity was reviewed by CDC, deemed not research, and was conducted consistent with applicable federal law and CDC policy.^§§^

## Preliminary Conclusions and Actions

In this community outbreak, five children developed measles. Although all patients were eligible to have received MMR vaccine before their exposures, none had been vaccinated because of cultural barriers, limited access to care, and vaccine refusal. Whereas previous measles outbreaks in the United States have primarily occurred in underimmunized communities with highly interconnected social networks (*1*), neither of the affected families described in this report was part of a similar close-knit social community, and vaccination coverage data for the patients’ sociocultural groups were not available. Public health responses have typically required tailored approaches that include developing culturally appropriate education materials, securing translation services, and building relationships with community leaders (*2*). These efforts are time-consuming and costly, with a median cost per measles patient of approximately $33,000 during 2004–2017 (*3*). This outbreak is a reminder that measles is highly contagious, and transmission can occur between children who are not social contacts. Outbreaks might become more common as global measles cases continue to rise (*4*) and the number of children with exemptions to childhood vaccines increases (*5*). Clinicians should consider measles in susceptible patients with febrile rash illness and clinically compatible measles symptoms. All eligible children and susceptible adults should receive 2 appropriately spaced doses of MMR vaccine to prevent measles and measles outbreaks.^¶¶^

## Footnotes

* The patient with clinically diagnosed measles was an extended family member of a similar age as the index patient. The exposure occurred in Yemen on September 24 or 25.

† All health care workers had either received 2 doses of MMR vaccine or had titers documenting immunity. One health care worker who had received 3 MMR vaccine doses was considered susceptible based on titer results; this worker was excluded from work and monitored for 21 days, during which time measles did not develop.

§ Immunity status was verified for 174 exposed patients and patient companions; three patients were lost to follow-up. Among the 174 patients with available information, 105 (60%) had documented proof of immunity (including two patients who received MMR vaccine within 72 hours of exposure). The remaining 69 (40%) persons were offered postexposure prophylaxis, if eligible; advised to quarantine (not attend work, school, or a child care facility); and were monitored for 21 days. None developed measles.

¶ Illinois state law mandates age-appropriate MMR vaccination for children attending schools and child care facilities; however, families with religious objections can obtain an exemption. None of the children in the family of the index patient were attending school or a child care facility at the time of the investigation. The director of the child care facility attended by patients D and E reported that a religious exemption form had been submitted for both unvaccinated children; however, despite multiple requests from CCDPH, copies of these forms were not provided.

** MMR vaccine was administered within 72 hours after exposure; immune globulin was administered within 6 days after exposure.

†† Measles patients are typically considered infectious from 4 days before through 4 days after rash onset.

§§ 45 C.F.R. part 46.102(l)(2), 21 C.F.R. part 56; 42 U.S.C. Sect. 241(d); 5 U.S.C. Sect. 552a; 44 U.S.C. Sect. 3501 et seq.

¶¶
https://www.cdc.gov/vaccines/vpd/mmr/public/
